# Sudden cardiac death due to intussusception of a coronary artery: a case report

**DOI:** 10.1007/s00414-025-03632-w

**Published:** 2025-10-17

**Authors:** Federica Attico, Francesco Di Paola, Matteo De Nadai, Gaetano Bulfamante, Andrea Verzeletti

**Affiliations:** 1 Institute of Legal Medicine of Brescia, University of Brescia, Piazzale Spedali Civili, 1, Brescia, 25123 Italy; 2 Department of Biomedical, Surgical and Dental Sciences, University of Milan, Milan, 20122 Italy; 3 Human Pathology and Molecular Pathology, TOMA Advanced Biomedical Assays S.p.A., Busto Arsizio, 21052 Italy

**Keywords:** Sudden cardiac death, Coronary artery intussusception, Spontaneous coronary artery dissection, Arterial fibromuscular dysplasia

## Abstract

In this case report, sudden cardiac death caused by intussusception of a coronary artery is discussed. A 47-year-old man was found dead in the nursing home where he lived, following an episode of polyphagia and two of vomiting. Upon cadaveric dissection, an overdistention of the large intestine was noted. Re-evaluation of the formalin-fixed whole heart revealed occlusion of the circumflex branch of the left coronary artery, which was not macroscopically attributable to vascular thrombosis or an atheromatous plaque. Histological investigations revealed ischaemic-type histological changes of the left ventricular wall in a hyperacute phase of evolution and, in the occluded coronary branch, extensive intraluminal invagination of the intima and media, as occurs in vascular intussusception. Further stains revealed the presence of fibromuscular dysplasia of the wall of the affected vessel. The subject’s death was ascribable to an acute cardiovascular failure secondary to acute ischaemic myocardial injury induced by intussusception of a coronary artery affected by dysplastic degeneration. These findings fully account for death by a mechanism sustained both by a mechanical deficit of the cardiac pump and by the possible onset of arrhythmias. Arterial intussusception is a rare complication of spontaneous coronary artery dissection. It is assumed that a combination of predisposing factors, which weaken the arterial wall, and trigger events, such as Valsalva-like activities, underlie the onset of the latter condition. This case highlights the importance of considering rare causes of sudden cardiac death. Greater awareness of these conditions can contribute to a more accurate identification of causes of death, with significant implications in both forensic and clinical settings.

## Introduction

Sudden cardiac death generally refers to a sudden death of a cardiovascular cause in an individual with or without pre-existing heart disease [1]. 

Coronary atherosclerosis is responsible for more than 50% of sudden deaths and for 90% of sudden cardiac deaths due to a coronary cause [2]. 

Among young subjects, the causes of sudden cardiac death of a coronary but non-atherosclerotic origin include anomalies in the origin, number and course of these vessels and the dissection of one or more of these. Spontaneous dissection of a coronary artery is a rare cause of sudden cardiac death. A rare consequence of this condition is intussusception, i.e. an invagination of a tubular wall into an adjacent portion of the same. Such a finding is reported in literature to affect the aorta, in a dissecting aneurysm and rarely in a coronary artery [3]. 

## Case report

We hereby present the case of a 47-year-old man suffering from obsessive-compulsive disorder and cognitive impairment, who had been living in a nursing home since the age of 26. These disorders were so severe as to make him completely dependent on others in the activities of daily living. The subject also suffered from epilepsy (well controlled with medication), high blood pressure and hypothyroidism. One afternoon, he manifested an episode of polyphagia – an expression of the obsessive-compulsive disorder from which he suffered – and consumed a considerable amount of jam. Some time later, he had two episodes of vomiting about two hours apart. The health personnel performed an enema, administered an antiemetic (metoclopramide) and checked his glycaemia, which was elevated (270 mg/dl).

Around midnight, the man appeared calm and the glycaemic control showed decreasing blood glucose values (124 mg/dl). At 4.30 a.m., the man was found lifeless by the nursing staff, who noted a very globose, tense and tympanitic abdomen as well as copious salivation and liquid vomiting.

The autopsy did not reveal any noteworthy external findings; upon cadaveric dissection, an overdistention of the large intestine, containing abundant pultaceous faecal matter, was noted. Upon dissection of the thorax, the pericardium was intact and free of adhesions. The heart was normal in shape and volume, weighing 360 g (in a 190 cm tall subject weighing 85 kg with a Body Mass Index of 23.5 Kg/m^2^ and a Body Surface Area of 2.11 m^2^). The transverse diameter measured 8 cm, and the longitudinal diameter measured 10.5 cm. A transverse cut was made along the short axis to allow for better fixation in formalin and to enable evaluation of the ventricular wall thickness: the free wall of the left ventricle measured 1.1 cm, the right ventricular wall measured 0.4 cm and septum measured 1.1 cm (all of these values are within the ranges of normality) [4]. 

Given the suddenness of the death and the absence of useful elements to identify its cause, it was deemed best to take the entire heart for evaluation following its fixation in formalin.

The formalin-fixed heart was re-evaluated by dissection according to the inflow-outflow method, with preservation of the main components of the cardiac conduction system. The coronary arteries were then evaluated by serial sectioning, with cuts every 3 mm, each one transverse to the major axis of the vessel, in accordance with the guidelines in cases of sudden death [5]. 

**Fig. 1:**
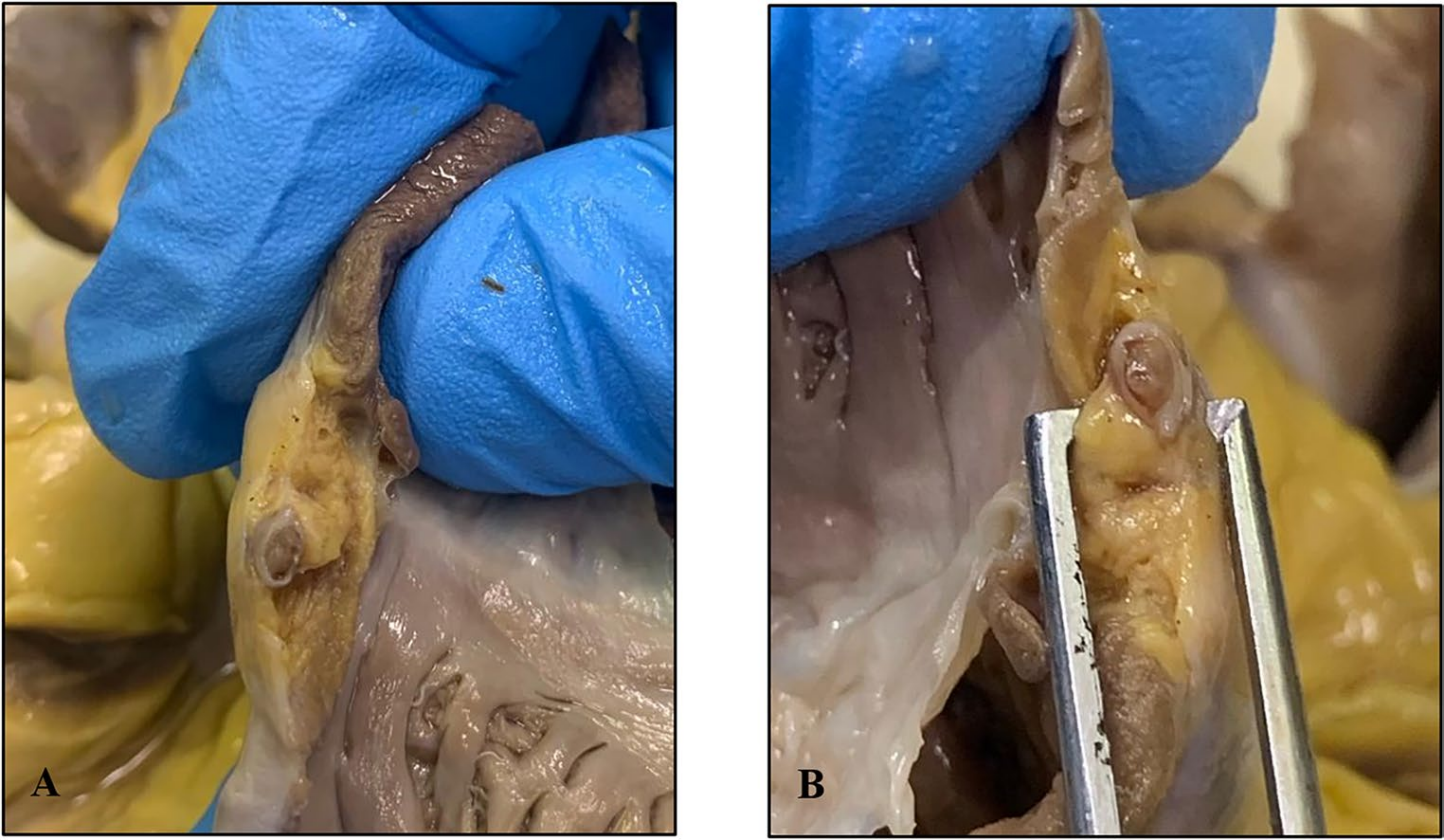
**A** and **B**: gross finding of the occlusion of the circumflex branch of the left coronary artery

The dissection of the coronary arteries revealed an occlusion extending for two of the transverse cuts of the proximal portion of the circumflex branch of the left coronary artery. The material occluding the lumen had a consistency which was similar to that of the vessel wall itself (Fig. 1). No other structural anomalies were detected affecting other coronary arteries, atria, ventricles, septa and heart valves.

For histological investigations, preparations of organ samples removed at autopsy (brain, lung, kidney, colon, small intestine and liver) stained with haematoxylin and eosin were prepared. Preparations of the myocardium and the circumflex branch of the left coronary artery stained with different dyes were then prepared.

**Fig. 2:**
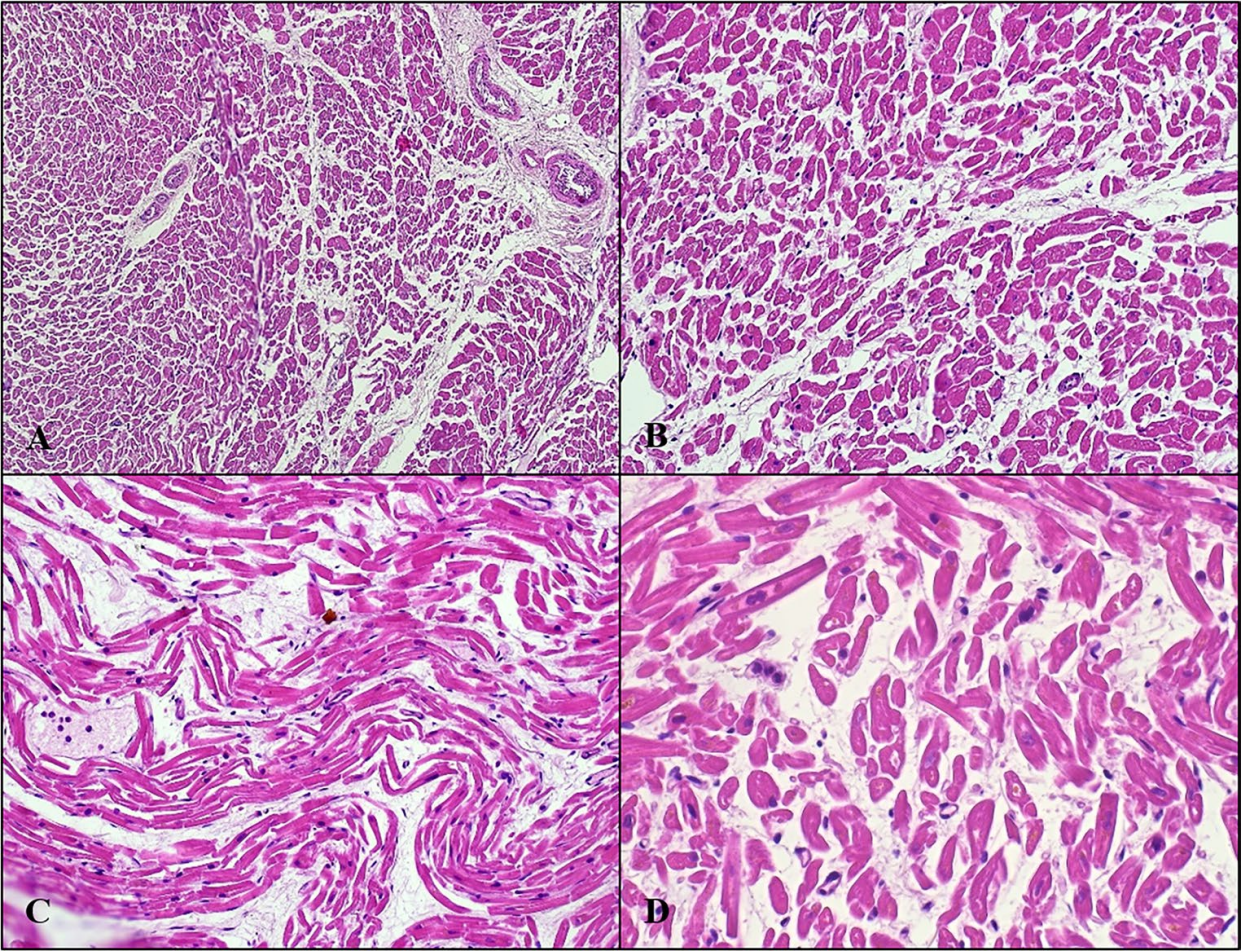
Left ventricular free wall: ischaemic-type histological changes in hyperacute phase of evolution. **A-B**. Interstitial oedema of cardiac wall; segmental expression. **C-D**. Undulation and dwindling of the myofibres are visible. Hematoxylin-Eosin stain

Histological investigations revealed, in the samples from the myocardium of the left ventricular free wall, a modest increase in subepicardial adipose tissue and, in the innermost third of the wall, ischaemic-type histological changes in a hyperacute phase of evolution (Fig. 2).

**Fig. 3:**
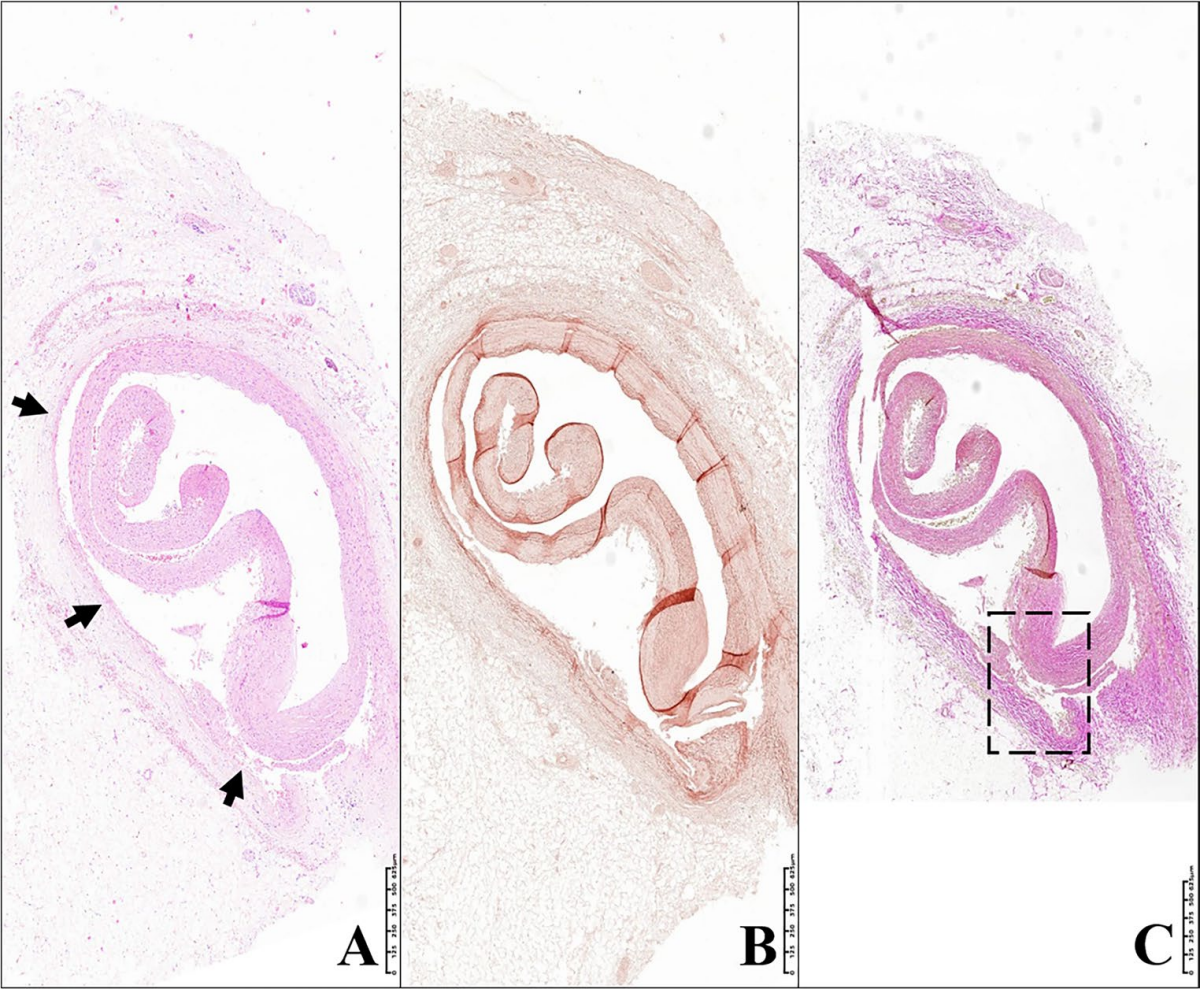
Circumflex branch of the left coronary artery. The blood vessel is histologically characterised by a partial tear in the tunica media (arrows). The dashed box in image C corresponds to figure n° 4. **(A)** Hematoxylin-Eosin stain; 3.10x. **(B)** Orcein stain; 3.30x. **(C)** van Gieson’s trichrome stain; 2.70x

**Fig. 4:**
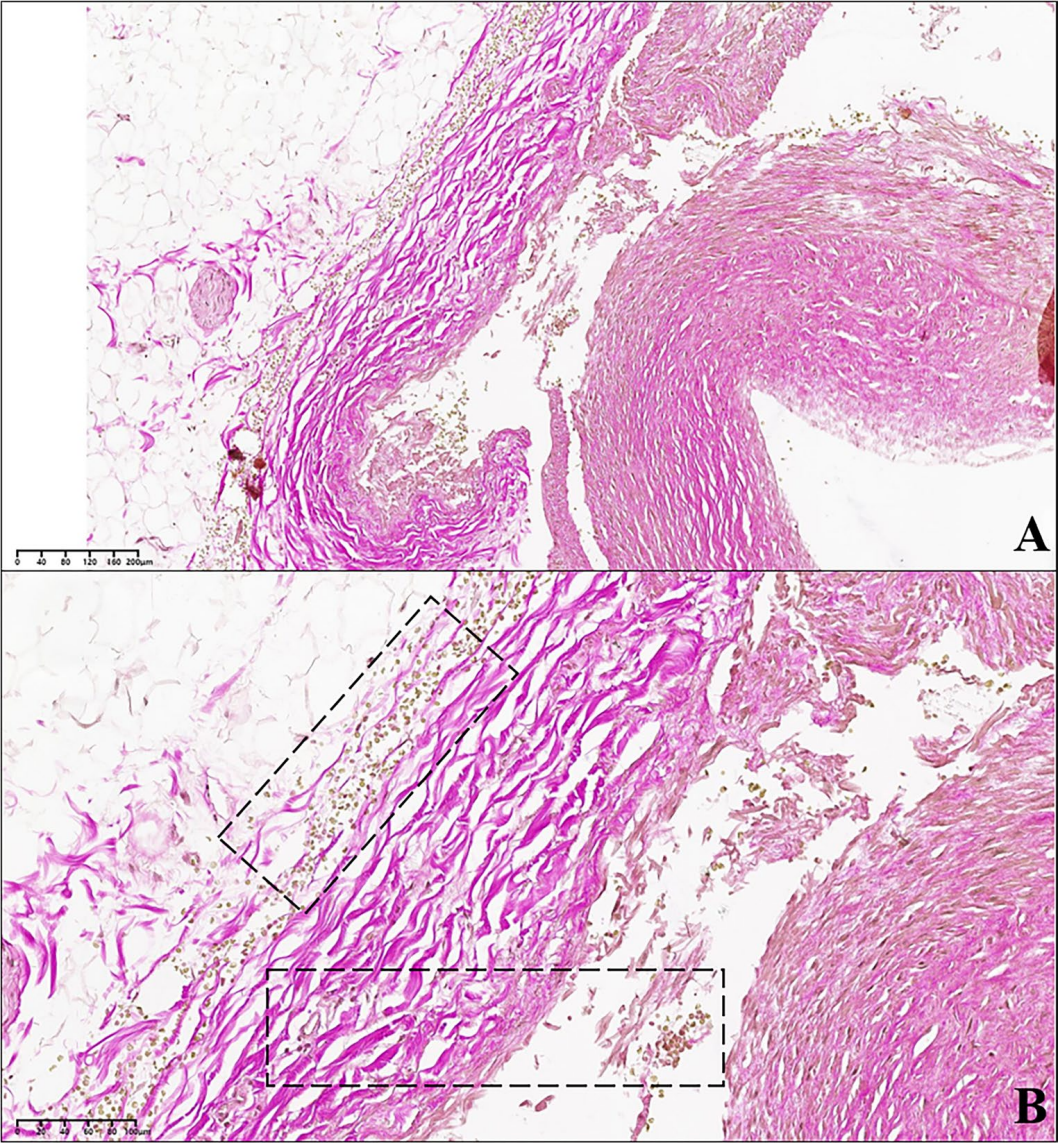
**A** and **B**: circumflex branch of the left coronary artery, at the level of the intussusception area. **(A)** Detail of an area of the arterial wall affected by dissection, which occurred at the interface between tunica media and tunica adventitia. **(B)** Detail at higher magnification of image «A». Numerous red blood cells (coloured yellow) can be observed in both the tunica adventitia and the space between adventitia and media; the dashed boxes indicate some areas occupied by erythrocytes (van Gieson’s trichrome stain; **A**: 10.80x; **B**:21.60x)

**Fig. 5:**
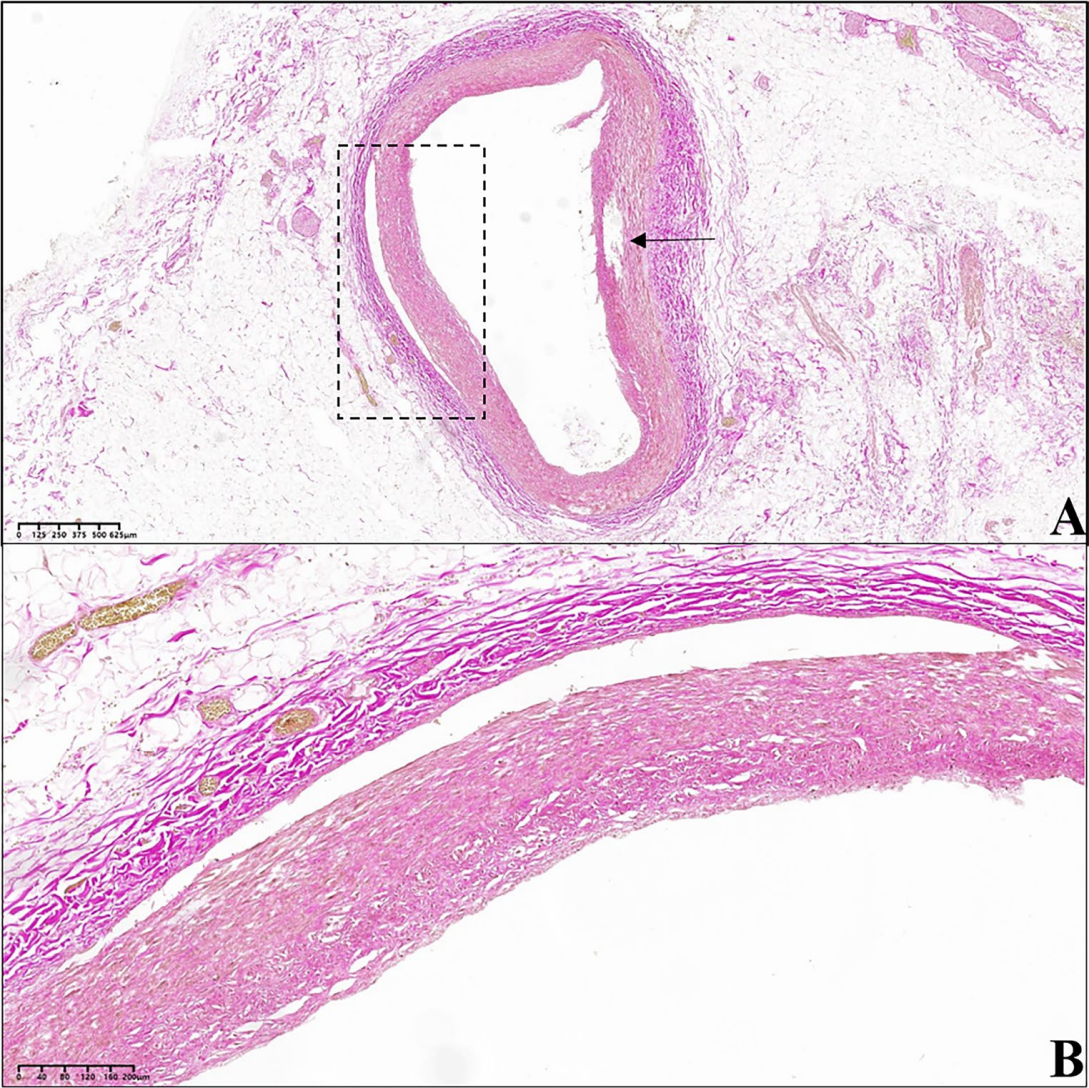
Another segment of the circumflex branch of the left coronary artery, in proximity of the intussusception area. **(A)** In the dashed box the vessel wall appears fissured for a tract shorter than that affected by vascular intussusception. The detachment edges are clear. The arrow identifies an area of the wall which was torn due to iatrogenic damage made while cutting the histological section. **(B)** Detail at higher magnification of the area in image «A», indicated in the dashed box. The vessel wall appears fissured at the junction between the tunica media and the tunica adventitia (van Gieson’s trichrome stain; **A**: 2.70x; **B**: 10.30x)

**Fig. 6:**
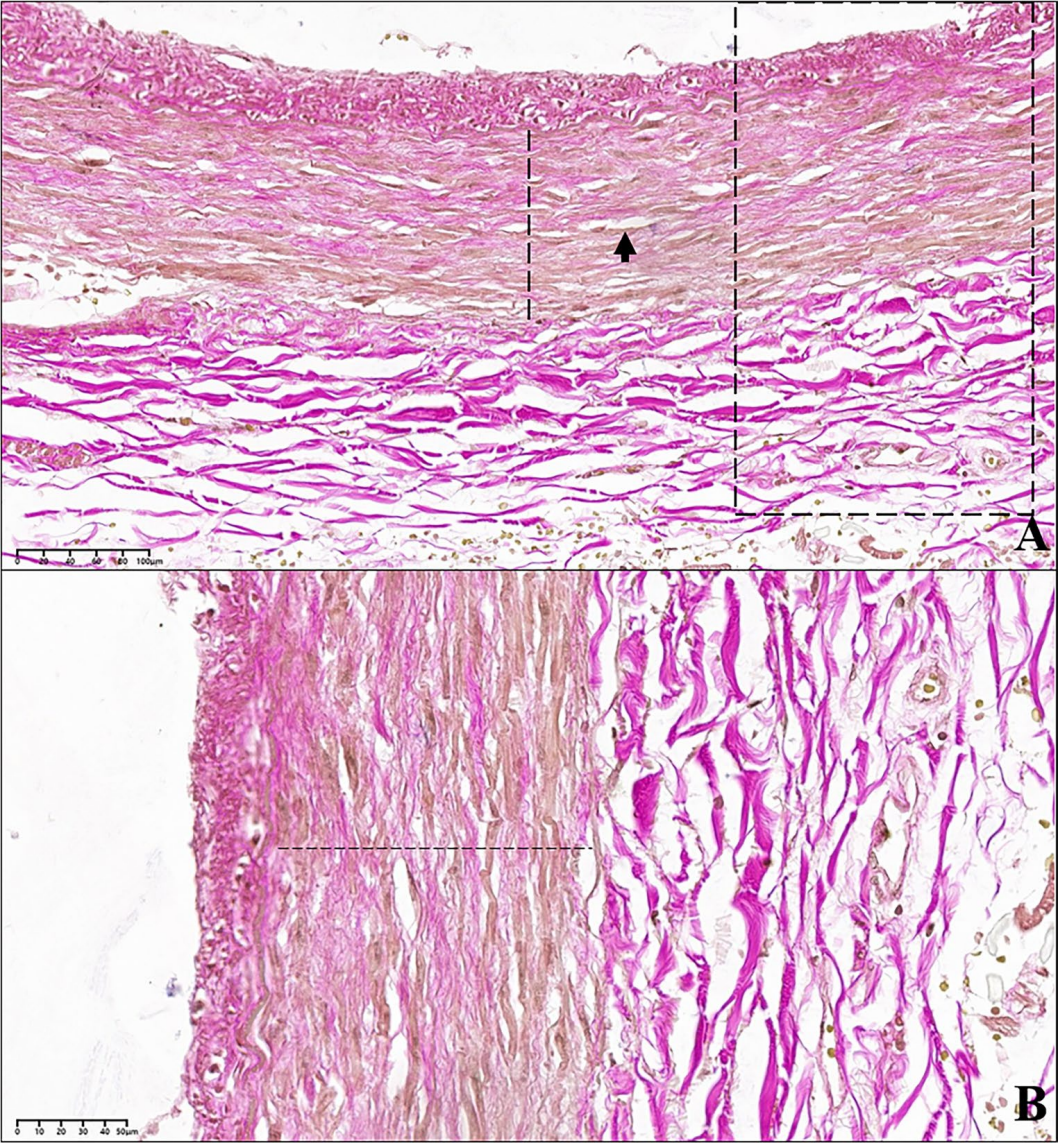
Circumflex branch of the left coronary artery, at the level of the intussusception area. **(A)** The tunica media (dashed line) is characterised by numerous small linear fissures arranged on a plane parallel to the endothelial one (arrow); van Gieson’s staining demonstrates how a part of the muscle fibres (coloured yellow) is substituted by connective tissue fibres (coloured purple). The dashed box indicates the area magnified in image (**B**) B. Detail at higher magnification of the dashed area in image «A». The connective tissue fibres (purple) are clearly widespread among the muscle fibres (yellow). The dashed line indicates the tunica media; below the latter, the arterial adventitia can be observed (van Gieson’s trichrome stain; **A**: 24x; **B**: 40x)

In the histological specimen prepared with haematoxylin and eosin from the circumflex branch of the left coronary artery, an arterial lumen occupied by intraluminal invagination of the tunica intima and media – the latter at times incomplete – was appreciated, thus delineating the entity of vascular intussusception. At the detachment areas, the presence of extravasated erythrocytes was observed, which were also present on the external perimeter of the tunica adventitia (Figs. 3 and 4). The vascular intima was only minimally thickened. The tunica media appeared linearly detached from the adventitia in some sections (Fig. 5), while in others, it was fissured linearly within itself (with frayed edges) on a plane parallel to the endothelial one (Fig. 6).

**Fig. 7:**
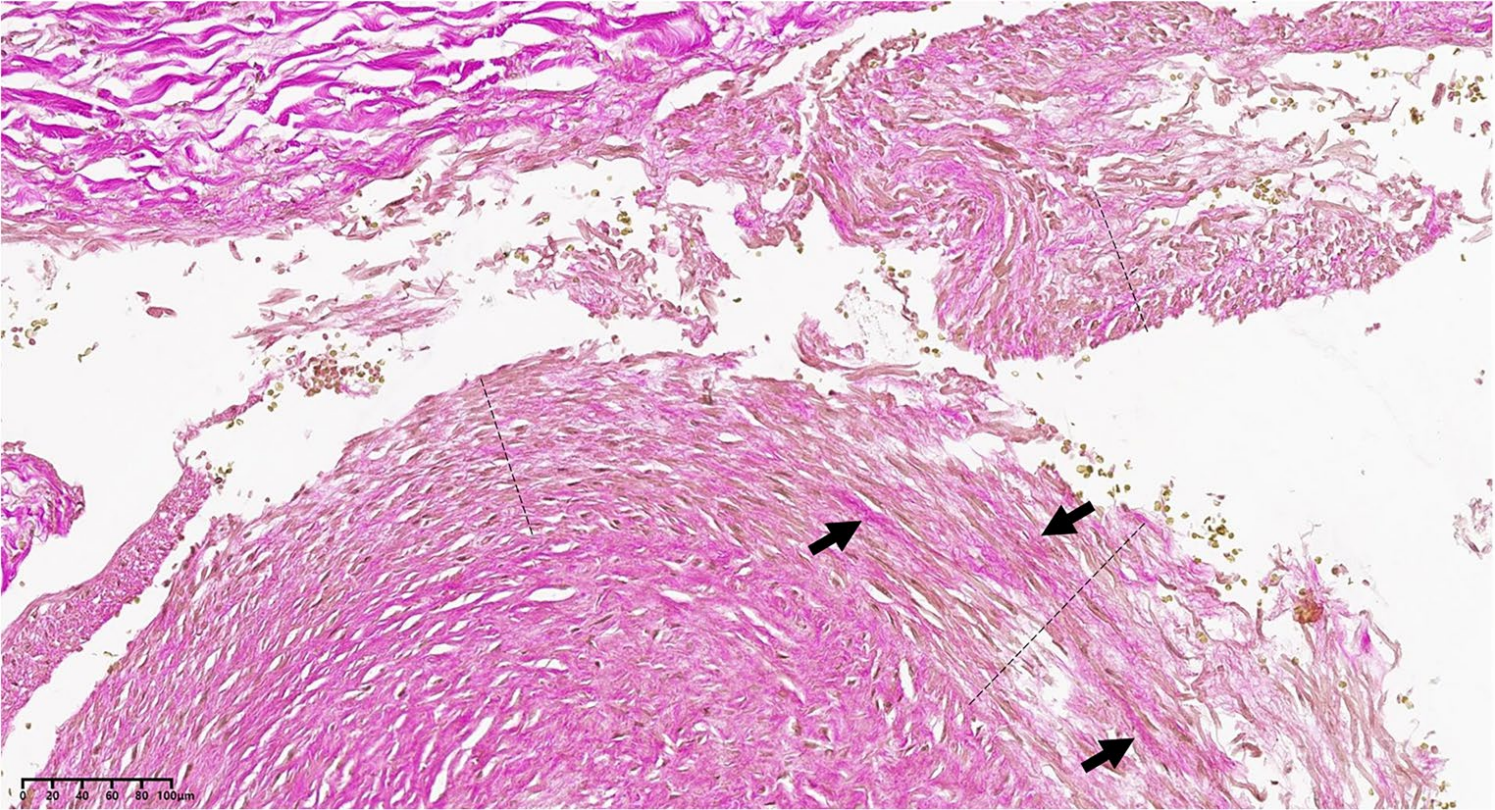
Circumflex branch of the left coronary artery, at the point of intussusception; the dashed lines indicate the vascular tunica media; on the top left, the tunica adventitia can be observed, composed of gross, purple-coloured connective tissue fibres. The tunica media appears interrupted and detached from the adventitia. The arrows indicate some gross connective tissue fibres (purple) within the media, extensively replacing the muscle fibres (yellow) (van Gieson’s trichrome stain; 19x)

**Fig. 8:**
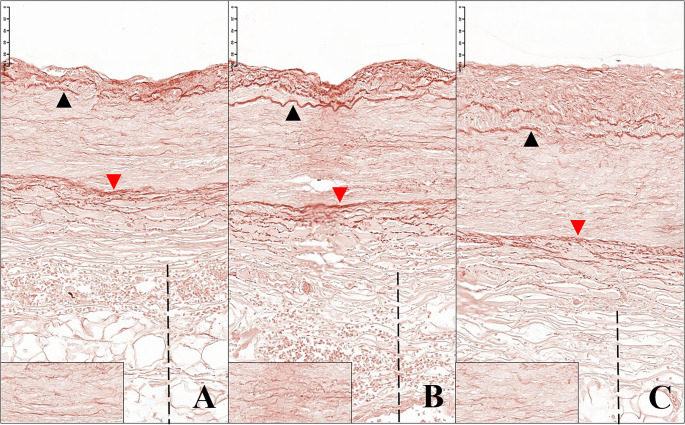
Circumflex branch of the left coronary artery. The elastic fibres are coloured dark brown. The black arrows indicate the inner elastic lamina of the artery, the red arrows the outer elastic lamina. The dashed line indicates the perivascular adipose tissue. The bottom left box highlights a detail of the thin elastic fibres which can be found within the vascular tunica media. **A-B.** Arterial tract affected by intussusception of its wall. The inner elastic lamina is often interrupted and appears doubled, and the thin underlying elastic fibres are fragmented and irregularly distributed. **C.** Segment of the same vessel in proximity of the intussusception area. Also in this portion the structure of the elastic component of the arterial wall appears abnormal (Orcein stain; **A**-**B**: 27x; **C**: 27,20x)

Van Gieson’s trichrome staining demonstrated the reduction of muscle fibres (coloured yellow) in the tunica media and their replacement by connective fibres (coloured red) (Fig. 7). The staining for elastic fibres revealed the reduction of elastic fibres in the vascular wall: both the inner and outer elastic laminae were modestly thinned and, in particular the inner one, often frayed and less compact than normal; in the tunica media, interposed between the two main elastic laminae, single elastic fibres were observed, which however, appeared rarefied and often fragmented into small segments (Fig. 8).

Trichrome van Gieson staining and staining for elastic fibres revealed the presence of fibromuscular dysplasia of the wall of the circumflex branch of the left coronary artery according to the American Heart Association classification system [6]. 

PTAH staining for fibrin ruled out the presence of vascular thrombosis, both recent and not, in all the samples examined (lungs, large intestine, small intestine, mesenteric blood vessels); these samples were chosen because the partial lysis of the circulating red blood cells cast diagnostic doubt on the presence of initial endovascular thrombotic phenomena.

All the blood vessels that were examinated appeared structurally normal.

On the basis of all the elements described thus far, in according with AECVP [7], it was possible to ascribe the subject’s death to an acute myocardial ischaemia induced by intussusception of the circumflex branch of the left coronary artery, which was affected by fibromuscular dysplasia.

Invagination of the vascular wall of the circumflex branch of the left coronary artery caused its occlusion and resulted in extensive ischaemic damage. These pathological findings fully account for death by a mechanism sustained both by a mechanical deficit of the cardiac pump and by the possible onset of arrhythmic events.

## Discussion

**Fig. 9:**
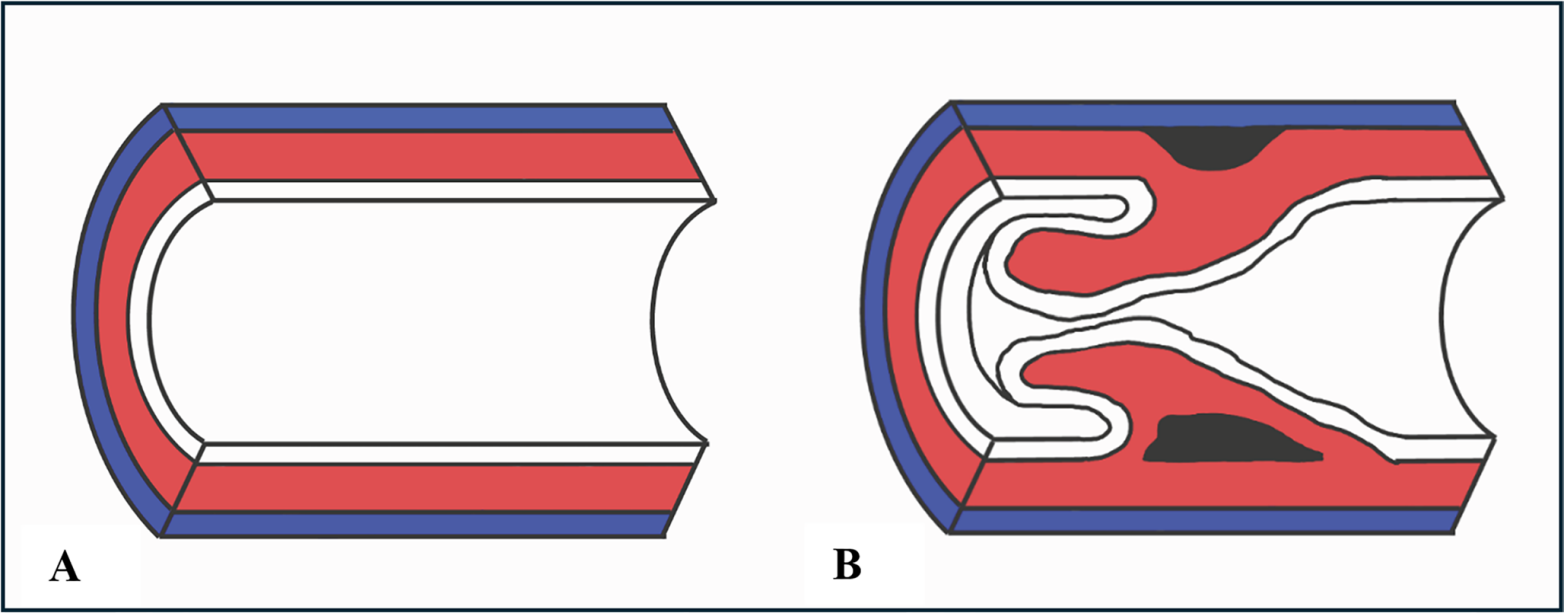
(**A**) Normal muscular arterial vessel: in blue, the tunica adventitia; in red, the tunica media; in white, the tunica intima. (**B**) Intussusception of a muscular arterial vessel: the media can partially detach (black space above) from the adventitia or rip apart within itself (black space below)

Vascular intussusception of a coronary artery is a rare pathological finding and it consists of invagination of the intima and, to a variable degree, of the media within the lumen of the vessel itself. In particular, the media may partially detach from the adventitia or rip apart within itself; the detachment area makes the affected tunicae more mobile, so that these herniate within their own vascular lumen, thus occluding it (Fig. 9).

This invagination of the vascular wall causes a sudden cessation of blood flow within the affected branch.

Arterial intussusception is most frequently a consequence of endovascular surgical procedures but is a rare finding when associated with another rare coronary artery condition, namely spontaneous dissection of the coronary arteries.

Spontaneous coronary artery dissection (SCAD) refers to the non-traumatic and non-iatrogenic development of a false lumen in the context of a coronary artery wall, unrelated to atherosclerotic disease or a primary dissection of the aorta [8]. 

Although the pathophysiological mechanism underlying SCAD is unknown, it is assumed that a combination of predisposing factors, which weaken the arterial wall, and trigger events underlie the onset of this condition [9]. 

Known predisposing factors include chronic inflammatory diseases (systemic lupus erythematosus, inflammatory bowel disease, coeliac disease, vasculitis-associated diseases, sarcoidosis, rheumatoid arthritis), connective tissue diseases (such as Marfan syndrome and Loeys-Dietz and Ehlers-Danlos type 4 syndrome) and conditions related to hormonal changes.

Arterial wall changes during pregnancy or during the use of hormonal therapies are well documented in literature, thus explaining the high incidence of female subjects with spontaneous coronary artery dissection in the puerperium [10]. 

Another predisposing condition is fibromuscular dysplasia, a non-atherosclerotic and non-inflammatory arterial wall disease, which appears to be the most frequent arteriopathy associated with SCAD [8]. 

Trigger events can be subdivided into emotional and physical, for example, intense physical stress, pregnancy and childbirth, Valsalva-like manoeuvres and recreational drug use [9]. 

The clinical presentation of SCAD is the same associated with acute coronary syndrome, with some subjects coming to medical attention for ventricular arrhythmias (< 10%), cardiogenic shock (< 3%) or sudden death (< 1%) [9]. 

In the case under discussion, the subject suffered from coronary fibromuscular dysplasia. From the circumstantial data, it was possible to identify in the intestinal distension and vomiting episodes, an intense Valsalva-like physical stress that is highly likely to have led to the dissection of the circumflex branch of the left coronary artery and the subsequent intussusception of the same.

## Conclusions

The case we outlined highlights the importance of taking into account even the less common conditions – which may be underestimated in forensic practice – when dealing with sudden cardiac death. The revealed pathological entity – i.e. coronary intussusception following spontaneous coronary artery dissection – appears to be rarely described in literature. The rarity of this condition and the possible overlap with other diseases may lead to disregard the *primum movens* of a sudden cardiac death. It is therefore crucial to perform a careful forensic analysis in cases of sudden cardiac death, especially in absence of obvious risk factors or preexisting heart disease. Greater awareness of these conditions can contribute to a more accurate identification of causes of death, with significant implications in both forensic and clinical settings.
